# Neural Network Connection Characteristics Between Motor Cortex and Spinal Cord During Rat Bipedal Walking

**DOI:** 10.1002/cns.71160

**Published:** 2026-09-18

**Authors:** Pengcheng Xi, Xiaodan Lyu, Jiping He, Rongyu Tang, Yiran Lang

**Affiliations:** ^1^ School of Mechatronical Engineering Beijing Institute of Technology Beijing China; ^2^ School of Life Science Beijing Institute of Technology Beijing China; ^3^ Institute of Automotive Engineers Hubei University of Automotive Technology Shiyan China; ^4^ Institute of Semiconductors Chinese Academy of Sciences Beijing China

**Keywords:** beta‐band oscillations, corticospinal functional connectivity, graph analysis, local field potential, network delay, spike train

## Abstract

**Object:**

Bipedal locomotion requires coordinated corticospinal interactions, yet how these networks dynamically reorganize across gait phases remains poorly understood. This study aimed to characterize the phase‐dependent restructuring of corticospinal functional networks during rest, stance, and swing in healthy rats.

**Methods:**

Single‐unit spiking activity was simultaneously recorded from the hindlimb motor cortex and lumbar spinal cord of healthy rats across three behavioral states: Rest, stance, and swing. Granger causality analysis was applied to local field potentials (LFPs) to assess directed functional connectivity. Total Spiking Probability Edges (TSPE) algorithm was used to estimate spike‐train functional connectivity. Network topology was characterized by global efficiency and rich‐club coefficient.

**Results:**

Granger causality revealed that corticospinal connectivity was most prominent in the beta band (13–35 Hz) during active locomotion. Global efficiency rose from rest (0.33) to stance (0.53) and swing (0.51). TSPE confirmed rich‐club topology in all conditions. Spinal hub neurons predominated during locomotion, comprising 81.13% ± 15.5% of rich‐club hubs at stance and 60.48% ± 10.21% at swing, while cortical and spinal hubs were balanced at rest. Mean latency decreased from 29.60 ± 20.66 ms at rest to 9.67 ± 1.65 ms during stance.

**Conclusions:**

The central nervous system dynamically restructures corticospinal functional networks across gait phases, with spinal hub neurons assuming a dominant role during active locomotion. This framework provides a mechanistic reference for studying corticospinal disruptions in spinal cord injury, Parkinson's disease, and stroke, and identifies phase‐specific beta‐band coupling and rich‐club hub dynamics as candidate targets for closed‐loop neural interfaces in gait rehabilitation.

## Graphical abstract

Our analyses revealed that the structure of the corticospinal network undergoes rhythmic reorganization during locomotion. The neural activities were recorded from the cerebral cortex and lumbar spinal cord segments of rats during bipedal walking on a treadmill. Through analyzing the low‐frequency neural signals, local field potentials, the interactions between the cortex and spinal cord were more prominent in the beta frequency band, while neuronal energy was more concentrated in the gamma band during movement. Using implantable multichannel electrode arrays, we sorted single‐neuron activity from both the motor cortex and spinal cord. Cross‐correlation analyses of spike trains were performed to estimate effective connectivity between the two regions. Our analyses—including assessments of neural activity patterns, network topology, node centrality, and signal transmission latency—revealed that the structure of the corticospinal network undergoes rhythmic reorganization during locomotion. Specifically, Rich‐club organization emerged in both the motor cortex and spinal cord, with these regions forming the primary hubs of connectivity. These results suggest that the central nervous system dynamically restructures its functional networks to accommodate different locomotor phases, with the spinal cord playing a key role in regulating bipedal walking.

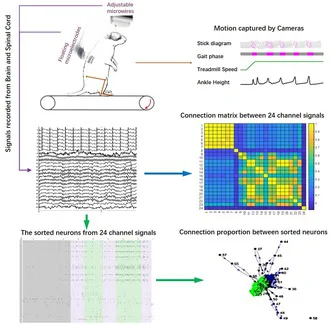

## 1 Introduction

Functional network connections between neurons are essential for enabling specific motor behaviors. Understanding these networks within the central nervous system (CNS)—encompassing the brain and spinal cord—is fundamental to explaining how coordinated locomotion is generated and controlled. The descending motor pathways form complex neural circuits involving the motor cortex, subcortical locomotor regions, medullary reticular formation, and spinal cord. Elucidating the functional connectivity between the motor cortex and spinal cord carries significant implications for both systems neuroscience and clinical neurorehabilitation.

Neuroimaging approaches, particularly functional MRI (fMRI), have substantially advanced our understanding of large‐scale connectivity in the brain and spinal cord [1, 2, 3, 4, 5]. For example, Kinany et al. [6] demonstrated fine‐grained resting‐state organization within the human spinal cord using fMRI, and rodent studies have characterized how spinal network architecture changes following injury [7, 8]. However, fMRI faces fundamental limitations that constrain its utility for studying corticospinal dynamics during active locomotion. Its spatial resolution (~1 mm) is insufficient to resolve connectivity at the level of individual neurons—the scale at which motor commands are ultimately encoded. Its temporal resolution (on the order of seconds) cannot capture the millisecond‐scale dynamics of corticospinal communication during movement. Moreover, susceptibility artifacts and the small cross‐sectional area of the spinal cord further degrade signal quality within spinal gray matter. These constraints make it impossible to determine how single cortical and spinal neurons interact in real time across distinct gait phases, motivating the use of invasive multichannel electrophysiology as an essential complementary approach.

Single‐neuron recordings offer a more granular perspective on network dynamics that imaging cannot provide [2, 9]. Combined electrophysiological studies of the spinal cord have revealed distinct functional architectures across its segments [10]. Notably, corticospinal neurons form postsynaptic connections with diverse spinal circuits, supporting both discrete and coordinated limb movements [11]. Furthermore, patch‐clamp evidence suggests that motor training actively remodels intracortical connectivity and strengthens descending communication to the spinal cord [12]. Together, these findings underscore the importance of characterizing corticospinal networks at the single‐cell level—both in healthy subjects and in the context of neural rehabilitation. However, existing studies have largely examined the cortex or spinal cord in isolation, leaving the dynamic interplay between these regions during ongoing locomotion poorly understood.

Over the past decades, multichannel extracellular recording technologies have enabled chronic monitoring of single‐unit activity in freely behaving animals. High‐density microelectrode arrays allow simultaneous recording from multiple neurons across brain regions [13, 14, 15, 16], providing rich datasets for studying neural population coding [17, 18, 19, 20, 21, 22]. The central pattern generator (CPG) in the spinal cord is a key regulator of rhythmic locomotion [23, 24, 25], and spontaneous and stimulation‐induced rhythmic output has been documented even in injured human spinal cord [26, 27], highlighting spinal circuits' intrinsic generative capacity. Nevertheless, relatively few studies have used invasive multielectrode recordings to directly characterize the functional interactions between motor cortex and spinal cord neurons during locomotion.

To quantify these interactions, multiple connectivity estimation methods have been developed, including Granger causality, transfer entropy, and cross‐correlation‐based approaches [28]. Pioneering work by Perkel, Gerstein, and Moore [29] introduced the use of cross‐correlation to study interactions between synchronously firing neurons. However, these methods are primarily designed for continuous signals and may not optimally capture the sparse, non‐stationary statistics of single‐unit spike trains. The Total Spiking Probability Edges (TSPE) algorithm addresses this gap by computing normalized cross‐correlations between spike train pairs and applying convolution with adaptive window functions. Critically, TSPE directly estimates synaptic transmission delays, distinguishes excitatory from inhibitory interactions, and demonstrates strong robustness on short‐duration, low‐firing‐rate recordings. Graph‐theoretical analysis further enables quantification of network topology—including degree, global efficiency, and clustering coefficient [30, 31]. One key metric is the “rich‐club” coefficient, which identifies a core group of highly connected hub neurons that preferentially interconnect with one another, forming a backbone for efficient information integration. Disruption of rich‐club organization has been linked to motor disorders such as Parkinson's disease and spinal cord injury, making it a clinically meaningful network marker.

To directly assess corticospinal functional connectivity during locomotion, we implanted microwire electrodes to simultaneously record from the hindlimb motor cortex and lumbar spinal cord of free‐moving rats. Bipedal walking was specifically chosen as the behavioral paradigm because, unlike quadrupedal locomotion, it demands continuous corticospinal engagement for upright balance and weight‐bearing—more closely approximating the neural control demands of human gait. We analyzed corticospinal functional networks across three distinct states (rest, stance, and swing) using TSPE‐based connectivity estimation and graph‐theoretical analysis. To our knowledge, this is the first study to characterize dynamic corticospinal network topology at the single‐neuron level during bipedal locomotion. Also, it provided a normative framework with direct translational relevance for gait rehabilitation in patients with spinal cord injury, Parkinson's disease, and stroke.

## 2 Methods

### 2.1 Animals

Four adult male Sprague–Dawley (SD) rats were individually housed in a ventilated animal facility maintained at 25°C ± 2°C, with ad libitum access to food and water. After one week of acclimatization, the rats were trained to perform bipedal walking on a treadmill, and their body weights were maintained at 300 ± 50 g. Reporting adhered to the ARRIVE (Animal Research: Reporting of In Vivo Experiments) guidelines.

### 2.2 Surgery

Surgical procedures followed previously published protocols [32]. Briefly, rats were anesthetized using 1.5%–2.5% isoflurane delivered in oxygen‐enriched air. Small incisions were made to expose the skull and lumbar vertebrae. An adjustable 2 × 8‐channel microwire electrode (MWE; diameter: 25 μm; inter‐wire spacing: 210 μm; Kedou Brain‐Computer Technology Co., Suzhou, China) was implanted into the hindlimb representation of the primary motor cortex (M1), with coordinates determined according to the Paxinos and Watson Rat Brain Atlas (7th edition) [33]: AP: +1.25 to +3.75 mm, ML: 2.5 mm, DV: 1.5–2.0 mm relative to bregma. Signal quality was monitored intraoperatively by assessing spontaneous multi‐unit activity, and electrode depth was adjusted in 20 μm increments until clear single‐unit waveforms were identified.

For spinal cord recording, a 2 × 4‐channel floating microelectrode array (fMEA; diameter: 25 μm; spacing: 400 μm; length: 4 mm; Kedou Brain‐Computer Technology Co.) was inserted at the L1–L2 vertebral segment [34]. Correct placement within the hindlimb motor circuitry was verified intraoperatively by confirming the presence of stimulus‐evoked hindlimb muscle responses (EMG) upon low‐current (≤ 50 μA) electrical stimulation through the electrode and by observing spontaneous multi‐unit activity patterns consistent with spinal interneuron firing.

Postoperative care included subcutaneous administration of enrofloxacin (5 mg/kg/day) for seven days to prevent infection, and buprenorphine (0.05 mg/kg, subcutaneous, twice daily) for three days to manage postoperative pain. Animals were monitored daily for signs of distress, infection, or neurological deficit. Electrophysiological recording commenced no earlier than seven days after full behavioral recovery.

### 2.3 Experimental Setup

**FIGURE 1:**
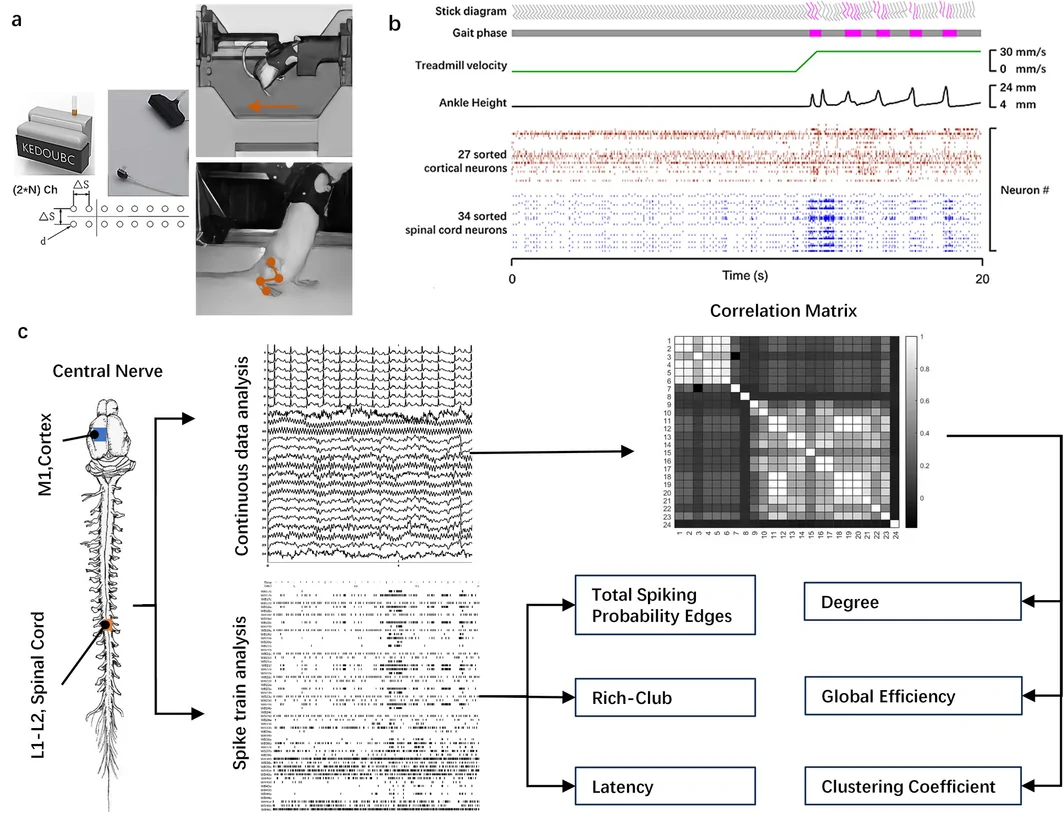
Kinematics and electrophysiological data of the cortex and the spinal cord from bipedal walking rodents. (a) Treadmill and rodent upper‐body setups supported the system, together with a chamber for forearm immobilization and distraction. Four hindlimb joints provided kinematic data. The movement of the hip, knee, ankle joints, and foot markers was tracked by a high‐speed motion‐capture system for gait analysis and phase definition. (b) Illustration of a transition moment from rest to walking. From top to bottom: Stick diagram of the joint connections, STANCE, and SWING phases (purple, SWING phase; gray, STANCE phase, and REST phase when the treadmill stopped), treadmill velocity, ankle joint height from the treadmill surface, raster plot of sorted cortical neurons (red), and spinal cord signals (blue). (c) Signal recording position and processing diagram. The adjustable multichannel array was mounted on the contralateral motor cortex of the hindlimb, according to the brain atlas. The floating microarray was inserted in the L1/L2 portion of the ipsilateral spinal cord.

Behavioral testing was performed on a computer‐controlled treadmill system (NeuroBehavior Inc., Beijing, China) with adjustable speed (1.5–3.5 cm/s) [35] (Figure 1a,b), trunk support height, and tilt angle. During training and experiments, rats wore a 3D‐printed jacket that restricted upper body movement and facilitated bipedal posture. A tranquilization chamber was placed in front of the treadmill to reduce stress. Training sessions lasted 10–20 min per day until rats could sustain continuous bipedal walking for at least 5 min. Each recording session lasted 3–5 min, followed by a 2‐min rest period. During each session, cortical, spinal, and kinematic data were recorded simultaneously.

### 2.4 Behavioral Analysis

Prior to recording, rats were fitted with a custom‐made vest and mounted onto the treadmill system. Reflective markers were attached to four hindlimb joints: Hip, knee (lateral condyle), ankle (lateral malleolus), and fifth metatarsophalangeal joint (MTP). A high‐speed camera captured joint motion, and the videos were analyzed offline using Simi Motion 2D software (version 9.2.2, SIMI Reality Motion Systems GmbH, Germany). The gait cycle was divided into stance and swing phases based on ankle contact and detachment from the treadmill belt. Rest state was defined as the period when the treadmill was stationary.

### 2.5 Electrophysiological Recording

Neural and kinematic signals were recorded using a Plexon data acquisition system (Plexon Inc., Dallas, TX, USA). Neural data were digitized at 40 kHz, and video was recorded at 80 frames per second.

For local field potential (LFP) analysis, signals were low‐pass filtered below 150 Hz and segmented into five standard frequency bands, as same as electroencephalogram: Theta (3–7 Hz), alpha (7–13 Hz), beta (13–35 Hz), gamma (35–50 Hz), and high gamma (50–150 Hz) [36]. Before signal analysis, raw signals were visually inspected, and epochs containing large‐amplitude transients (peak‐to‐peak amplitude > 5 × the baseline standard deviation within a 300 ms sliding window) were automatically flagged and excluded. To minimize EMG contamination in LFP signals, a spatial common‐average reference (CAR) was applied across channels within each array, and a 50 Hz notch filter was used to suppress power‐line interference. Granger Causality (GC) analysis was conducted using the HERMES MATLAB toolbox (v2020.04.26) to compute connectivity matrices. Topological properties [37], including degree, global efficiency, and clustering coefficient, were derived from the GC model. The clustering coefficient quantifies local connection density, while global efficiency reflects the overall communication efficiency of the network.

For spike sorting, signals were high‐pass filtered above 350 Hz. After that, waveforms with peak‐to‐valley amplitudes below 10 μV or with signal‐to‐noise ratios less than 3.5 were discarded as noise. Additionally, waveforms exhibiting shapes inconsistent with action potentials (e.g., absence of a clear negative peak, biphasic artifacts without a refractory period) were excluded by template matching in Plexon Offline Sorter (V4.3.1). To further verify signal stationarity during locomotion, electrode impedance was measured before and after each recording session; sessions with impedance changes greater than 20% were excluded from analysis.

### 2.6 Total Spiking Probability Edges (TSPE)

We applied the TSPE algorithm to assess functional connectivity between spike trains. TSPE computes normalized cross‐correlation (NCC) between pairs of spike trains and applies convolution with window functions to derive a smoothed similarity curve. The temporal delay between neurons corresponds to the peak location of the TSPE curve, and the peak amplitude reflects the strength of similarity. This method distinguishes excitatory and inhibitory interactions and estimates transmission delays with high accuracy. Detailed mathematical formulations of TSPE have been described previously [38].

The TSPE algorithm is as follows:

$$\begin{array}{c} {\mathrm{NCC}}_{x\to y\left(d\right)}=\frac{1}{N}\sum_{i=-\infty }^{\infty }\frac{\left(y\left(i\right)-y^{\prime }\right)\cdot \left(x\left(i-d\right)-x^{\prime }\right)}{\mathrm{\sigma x}\cdot \mathrm{\sigma y}} \end{array}$$

$${\mathrm{SPE}}_{x\to y\left(d\right)}={\mathrm{NCC}}_{x\to y\left(d\right)}*g\left(i\right)$$

$${\text{TSPE}}_{x\to y\left(d\right)}=\sum_{n=1}^{N_{a}\cdot N_{b}\cdot N_{c}}{\mathrm{SPE}\prime }_{X\to Y\left(d\right)}^{\left(n\right)}*h_{\left(i\right)}^{\left(n\right)}$$

where:

x: Spike train *x*.

y: Spike train *y*.

d: The temporal displacement.

NCC: The cross‐correlation between spike train *X* and spike train *Y*.

$g\left(i\right)$: 1D edge filter.

${\mathrm{SPE}}^{\prime }$: Each ${\mathrm{SPE}}_{x\to y\left(d\right)}$ is divided by the sum over all neuron pair results for the delay d.

$h\left(i\right)$: Running total filter.

### 2.7 Rich‐Club Organization

Rich‐club organization refers to the tendency of high‐degree nodes to form tightly interconnected clusters, serving as a hub for information exchange [39, 40]. We evaluated this property using the weighted rich‐club coefficient $\Phi^{\omega }\left(k\right)$:

$$\Phi^{\omega }\left(k\right)=\frac{W_{>k}}{\sum_{l=1}^{E>k}w_{l}^{\text{ranked}}}$$

where:

$W_{>k}$: Denotes the set of nodes with degree strictly greater than k.

$E_{>k}$: The total number of edges among nodes in $W_{>k}$.

$w_{l}^{\text{ranked}}$: Represents the l‐th largest edge weight in the entire network, used to normalize the numerator against the maximum possible weight that could be assigned to $E_{>k}$ edges.where nodes with degrees less than k are excluded, and the numerator sums the weights of the remaining edges. To assess whether the observed network exhibits rich‐club characteristics, we normalized $\Phi^{\omega }\left(k\right)$ by dividing it by the corresponding value from a randomized network $\text{Φrandom}\left(k\right)$:

$$\text{Φnorm}\left(k\right)=\frac{\Phi \left(k\right)}{\text{Φrandom}\left(k\right)}$$

A $\text{Φnorm}\left(k\right)$ value greater than 1 indicates the presence of a rich‐club structure. This analysis was applied to networks derived from rest, stance, and swing phases.

### 2.8 Statistical Analysis

All data are reported as mean ± standard error of the mean (SEM) for normally distributed variables. Prior to parametric testing, normality of each variable was assessed using the Shapiro–Wilk test (*p* > 0.05 indicating normality). For normally distributed data, within‐subject comparisons across locomotor phases were performed using paired *t*‐tests. For non‐normally distributed data or small‐sample comparisons, the Wilcoxon signed‐rank test was used as a non‐parametric alternative. Bonferroni correction was applied when two‐way ANOVA was used for analyzing the effect of states and frequency band on neuron connection. For FDR‐controlled analyses of network edges, the Benjamini–Hochberg procedure was used at *q* < 0.05. Linear regression was performed to examine relationships between network metrics and locomotor kinematics. All statistical analyses were conducted in SPSS (version 24, IBM Corp., USA) and MATLAB (R2021a, MathWorks).

## 3 Results

### 3.1 Functional Connectivity in Low‐Frequency Neural Signals

**FIGURE 2:**
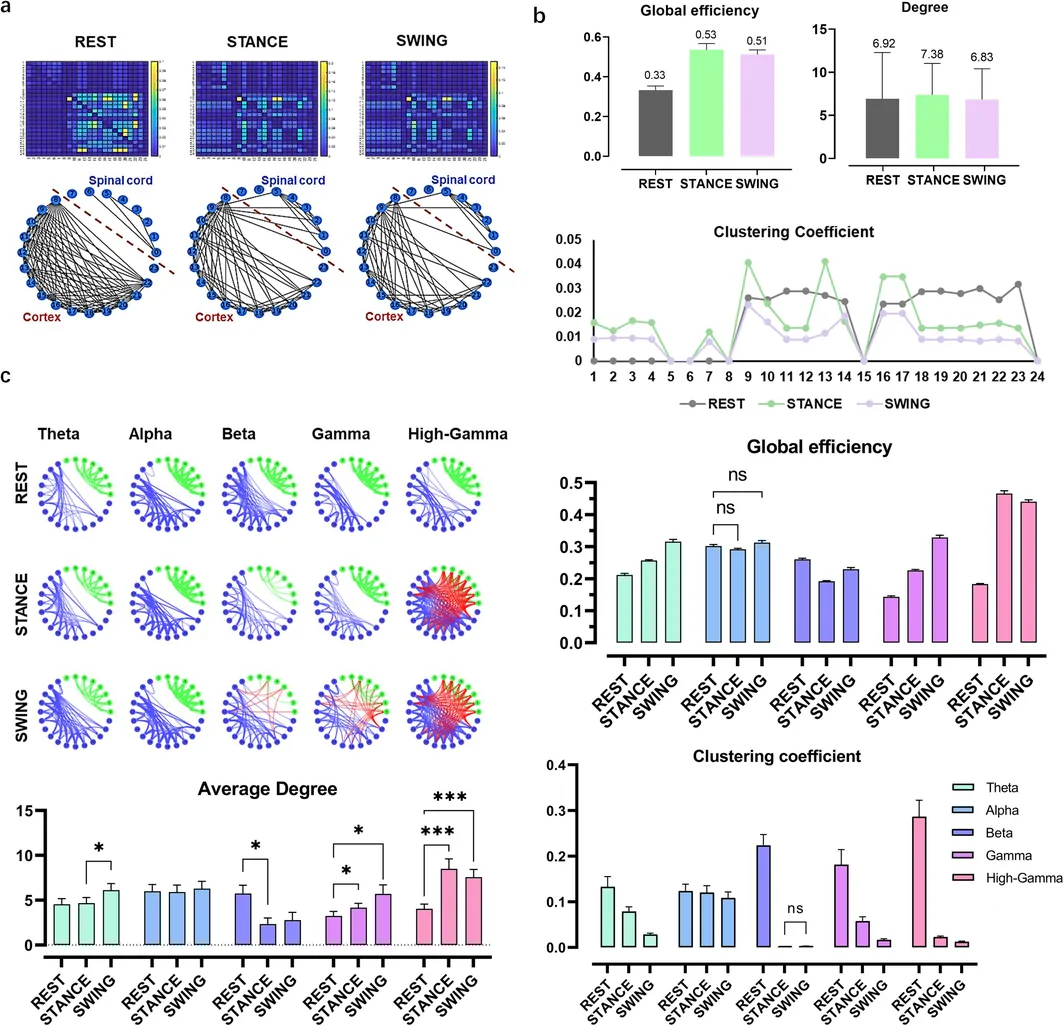
Connectivity matrix analysis in the low‐frequency band and subdivided periods. (a) Connectivity matrix and node connection diagram under low‐frequency neural signals; (b) the Global efficiency, Degree, and Clustering Coefficient calculated in the entire low‐frequency band; (c) the connection diagram, Global efficiency, Clustering Coefficient, and Average Degree in the subdivided frequency band.

To investigate corticospinal connectivity across different movement states, we first analyzed low‐frequency neural signals from the hindlimbs of rats during rest, stance, and swing phases (Figure 2a). The connectivity matrices and corresponding network topology parameters revealed significant differences in neural interactions between the motor cortex and spinal cord across these phases.

The network graphs clearly showed that corticospinal connectivity was stronger during the stance phase compared to the swing phase. Global efficiency was significantly higher during active locomotion than during rest stance: 0.53 ± 0.03 vs. rest: 0.33 ± 0.02, *p* < 0.01; swing: 0.51 ± 0.02 vs. rest: 0.33 ± 0.02, *p* < 0.01. In the subdivided frequency band analysis (Figure 2c), beta‐band global efficiency differed significantly across phases (two‐way repeated‐measures ANOVA: *F*(2,240) = 608.3, *p* < 0.001), with post hoc comparisons revealing significantly lower global efficiency in the beta band during rest compared to stance (*p* < 0.001), (Bonferroni‐corrected) and swing (*p* < 0.001). Node degree in the beta band was significantly higher during swing (2.97 ± 0.86) than during stance (2.33 ± 0.69; *t*([2]) = 3.39, *p* < 0.05). Statistical significance annotations (*, *p* < 0.05; **, *p* < 0.01; ***, *p* < 0.001; ns, not significant) are displayed directly on Figure 2c.

We further examined how connectivity varied across different frequency bands. Signal decomposition demonstrated that the beta band exhibited the strongest inter‐region (cortex‐to‐spinal cord) connectivity (Figure 2c), while the other frequency bands mainly reflected intra‐regional interactions. The high‐gamma band showed particularly dense local connectivity within both brain and spinal regions. Despite the stronger beta‐band connectivity, this band had the lowest global efficiency values, indicating that while specific pathways became prominent, the overall network was less integrated (Figure 2c). In terms of beta‐band connectivity, node degrees were 2.33 and 2.97 for stance and swing, respectively, while clustering coefficients were lowest across all phases. These results suggest that motor control during stance requires higher neuronal engagement. Individual animal data for all core network metrics (global efficiency, clustering coefficient) are provided in Figures S1–S9, demonstrating consistent phase‐dependent patterns across all four animals.

### 3.2 Single‐Unit Activity and TSPE‐Based Connectivity in High‐Frequency Signals

**FIGURE 3:**
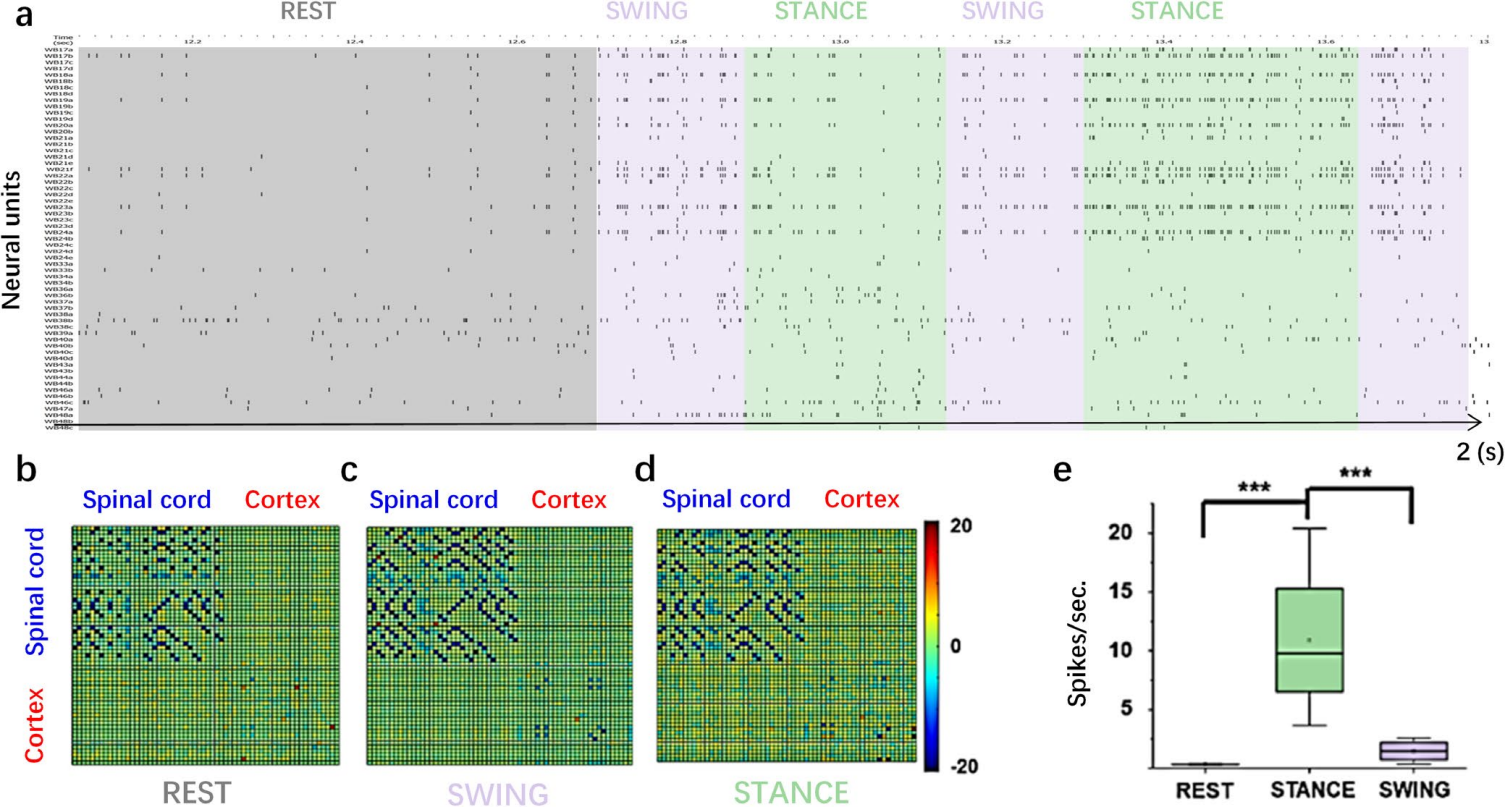
Neural Activity Dependence across Behavioral States. (a) Neural units in the REST state, STANCE, and SWING phases within 2 s. (b–d) Matrices of estimated connectivity amongunits. (e) The average number of spikes in each phase. Two‐way repeated‐measures ANOVA with Bonferroni correction.

High‐pass filtered signals were analyzed to assess discrete spiking activity during different behavioral states. In the present study, each animal contributed simultaneously recorded cortical and spinal single units across multiple sessions, yielding a total analytical dataset comprising 33 cortical units and 27 spinal units. Although average firing rates of cortical and spinal neurons were similar, their temporal activity patterns differed significantly between rest, stance, and swing phases (Figure 3a). Connectivity matrices generated using the Total Spiking Probability Edges (TSPE) algorithm revealed that spiking coordination varied across phases (Figure 3b–d).

At rest, the average firing rate was significantly lower (0.34 ± 0.049 spikes/s per unit) compared to the stance phase (10.89 ± 6.97 spikes/s, *p* = 0.005). The swing phase showed an intermediate rate (1.45 ± 0.96 spikes/s), with no significant difference from rest (*p* = 0.708). However, firing rates differed significantly between stance and swing phases (*p* = 0.009, Figure 3e).

### 3.3 Rich‐Club Organization of the Corticospinal Network

**FIGURE 4:**
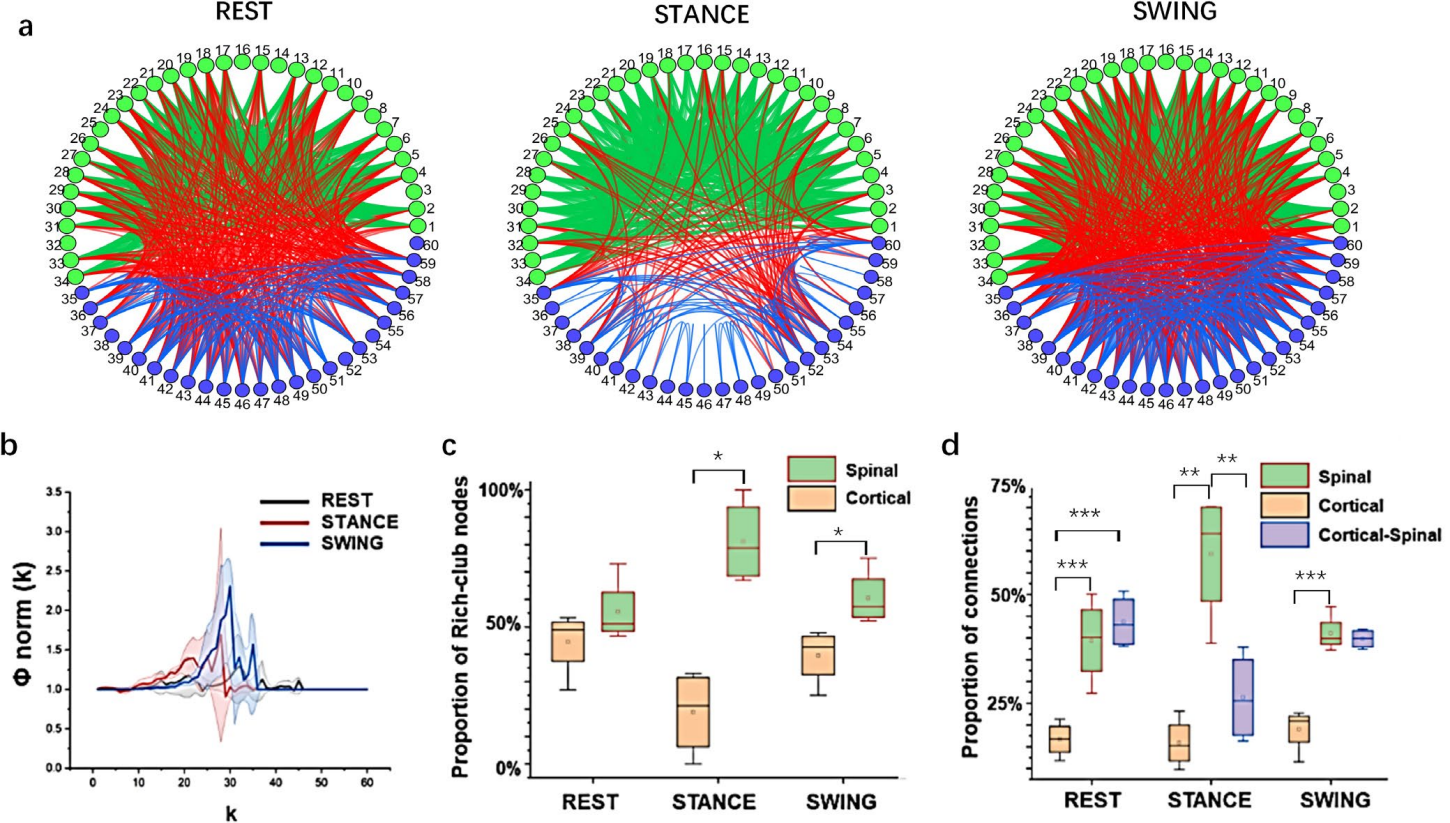
Analysis of connectivity and Rich‐Club property. (a) Connectivity map and isolated units in various phases of locomotion. Green dots: Cortical neurons. Blue dots: Spinal neurons. The units that were not involved in the network were depicted individually. (b) Distribution of the Rich‐Club index. The horizontal axis represents the node degree, and the vertical axis is the standardized Rich‐Club coefficient. (c) The proportion of Rich‐Club nodes across different phases. (d) The proportion of connections in different phases.

The TSPE‐based functional connectivity network was visualized across behavioral states (Figure 4a). During stance, nearly all cortical and spinal neurons (≥ 98%) participated in the network with more than one connection. In contrast, during rest and swing, approximately 25% of cortical nodes had fewer than two connections, suggesting a decrease in cortical involvement.

To directly address the central question of how locomotion reorganizes corticospinal hub structure relative to rest, we compared the proportion of spinal rich‐club nodes across locomotor states (Figure 4c). Spinal hub node proportion increased significantly from rest (55.48% ± 11.9%) to stance (81.13% ± 15.5%, *p* < 0.05) and from rest to swing (60.48% ± 10.21%, *p* < 0.05). The stance phase showed a significantly higher spinal hub proportion than swing, indicating a graded, phase‐dependent shift in network dominance toward spinal circuits during active weight‐bearing. Conversely, cortical hub node proportion showed a corresponding decrease from rest (44.53% ± 11.9%) to stance (18.88% ± 15.5%; *p* < 0.05) and a partial recovery during swing (39.53% ± 10.21%). These cross‐state comparisons are presented in the restructured Figure 4c, with individual animal trajectories overlaid to illustrate the consistency of the hub‐shift direction across all four animals. Furthermore, during rest, the number of intracortical connections was significantly lower than both intraspinal connections and corticospinal connections (*p* < 0.001). During stance, intraspinal connectivity increased significantly to 59.27% ± 14.77% (*p* < 0.05). In the swing phase, connection distributions returned to patterns resembling rest (Figure 4d). Individual animal data for all core network metrics are provided in Figures S10 and S11, demonstrating consistent phase‐dependent patterns across all four animals.

### 3.4 Information Transmission Latency Across Phases

**FIGURE 5:**
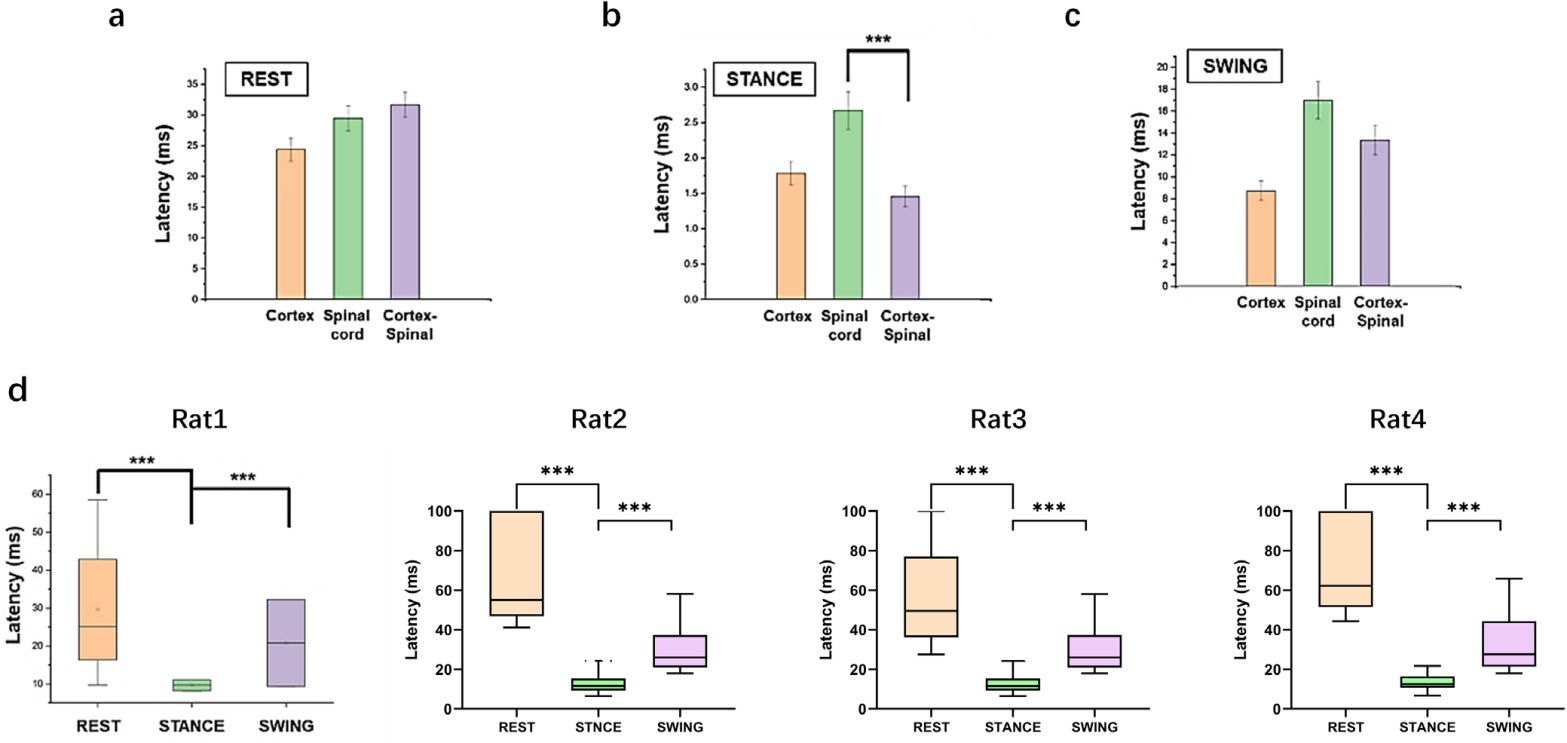
Analysis of network delay of information transmission. Comparison of network delay duration among different locomotion stages for (a) REST, (b) STANCE, and (c) SWING of rat 1. (d) Averaged delay box graph in four rats.

We also examined the latency of neuronal signal transmission within and between brain and spinal nodes. Overall network latency was highest during rest (29.60 ± 20.66 ms, median = 24.1 ms [IQR: 14.2–38.6 ms]), significantly reduced during stance (9.67 ± 1.65 ms, median = 9.2 ms [IQR: 7.8–11.4 ms], *p* < 0.001), and intermediate during swing (16.36 ± 13.24 ms, median = 21.2 ms [IQR: 9.6–32.4 ms], *p* < 0.05 vs. stance, Figure 5d).

Detailed comparisons of regional latency revealed no significant differences among cortical, spinal, and corticospinal transmission delays during rest, stance, and swing phases (Figure 5a–c). However, in the stance phase, intraspinal transmission latency was significantly longer than corticospinal (cortical‐to‐spinal) transmission latency (*p* < 0.01; Figure 5b), suggesting that once descending motor commands arrive at the spinal cord, information is rapidly relayed via direct corticospinal synaptic contacts, while intraspinal circuit processing involves additional interneuronal steps with longer cumulative delays.

## 4 Discussion

This study employed implantable multichannel electrode arrays to simultaneously record single‐unit activity from the hindlimb motor cortex and lumbar spinal cord of rats performing bipedal locomotion, enabling direct characterization of corticospinal functional network dynamics across rest, stance, and swing phases. Three principal quantitative findings emerged. First, network global efficiency increased substantially from rest (0.33) to stance (0.53) and swing (0.51), indicating that locomotion drives a more integrated corticospinal communication architecture. Second, the proportion of spinal rich‐club hub nodes rose from 55.48% at rest to 81.13% during stance (*p* < 0.05), demonstrating a locomotion‐dependent redistribution of network centrality toward spinal circuits. Third, mean signal transmission latency decreased from 29.60 ± 20.66 ms at rest to 9.67 ± 1.65 ms during stance (*p* < 0.001), reflecting accelerated inter‐regional signaling during active weight‐bearing. Together, these findings reveal that the corticospinal network undergoes rhythmic, phase‐specific reorganization during bipedal locomotion, with spinal neurons assuming a dominant hub role during the stance phase rather than functioning as passive relay elements.

### 4.1 Corticospinal Interactions in Beta and Gamma Bands

In our analysis, Granger causality estimates revealed that corticospinal interactions in the beta band (13–35 Hz) were most prominent during the stance phase. Directional analysis of GC showed that beta‐band effective connectivity was bidirectional but asymmetric, with cortex‐to‐spinal‐cord influence significantly stronger than the reverse spinal‐cord‐to‐cortex direction, suggesting that descending motor commands constitute the dominant information flow during the stance phase while proprioceptive feedback pathways also contribute to beta‐band corticospinal network dynamics. This is consistent with the established role of beta oscillations in corticospinal motor command transmission [41].

However, a direct comparison with the findings of Hao et al. [42] requires caution for two reasons. First, Hao et al. identified two distinct temporal roles of beta synchrony in human motor behavior: Pre‐movement corticospinal coordination and sustained synchrony during movement maintenance. Our observation of peak beta coupling during the stance phase—an active, sustained weight‐bearing state—is more consistent with the second role (movement maintenance) rather than pre‐movement preparation and is therefore not contradictory but rather phase‐selective. Second, Hao et al.'s findings were obtained in humans, whose corticospinal system differs from that of rats in the proportion of direct monosynaptic cortico‐motoneuronal connections (far more prevalent in primates than in rodents) [43]. The beta‐band coupling observed in the rat corticospinal system may therefore reflect polysynaptic relay dynamics rather than the direct cortico‐motoneuronal synchrony described in human studies. These species differences limit direct mechanistic equivalence and are acknowledged as an interpretive constraint.

Elevated gamma (35–50 Hz) and high‐gamma (50–150 Hz) power were observed in LFP signals from both the motor cortex and spinal cord during active locomotion compared to rest. While this pattern is consistent with a role for high‐frequency oscillations in rapid, fine‐tuned motor control [44], two interpretive cautions are warranted. First, high‐frequency LFP components in the spinal cord are particularly susceptible to contamination from electromyographic (EMG) signals and movement‐related mechanical artifacts, given the proximity of the recording electrodes to paraspinal musculature. Although spatial common‐average referencing and motion artifact rejection procedures were applied, residual EMG contamination in the gamma/high‐gamma range cannot be fully excluded. Future studies incorporating simultaneous EMG recording with independent component analysis (ICA)‐based artifact separation would strengthen the attribution of high‐frequency spinal LFP to local neural network activity. Second, the present experiment did not systematically vary treadmill speed or parse movement episodes by the degree of motor precision required, precluding a direct test of whether gamma/high‐gamma power scales with fine motor demands. The “fine‐tuned control” interpretation is therefore offered as a hypothesis consistent with the data, rather than a conclusion directly supported by the experimental design.

### 4.2 Corticospinal Signal Transmission Under Complex Network Structure

During the stance phase, spinal neurons exhibited denser rich‐club organization and shorter intraspinal transmission latencies than those during rest. However, within the stance phase, intraspinal transmission latency remained longer than corticospinal latency, as reported in Section 3.4. These findings suggest that spinal circuits may contribute more prominently to corticospinal network organization during active weight‐bearing. However, the mechanistic interpretation of this “spinal dominance” warrants careful qualification. The observed increase in spinal hub connectivity and network efficiency could reflect at least three distinct, non‐mutually‐exclusive mechanisms. First, enhanced sensory feedback during stance—including proprioceptive signals from muscle spindles and cutaneous input from plantar contact—is routed through dorsal horn and intermediate zone circuits, and could independently elevate spinal interneuronal activity and intra‐spinal connectivity without requiring increased descending motor drive [45]. Second, the intrinsic rhythm‐generating capacity of lumbar central pattern generator (CPG) circuits may be more strongly engaged during the weight‐bearing stance phase, producing autonomous bursting patterns that appear as high‐density intra‐spinal connectivity in network analysis but do not necessarily reflect descending cortical command transmission [34]. Third, genuine integration of descending motor commands with local spinal interneuronal circuits could account for the observed hub shift [46, 47]. The current data—derived from correlational network metrics rather than causal intervention—cannot distinguish among these mechanisms. We therefore describe the stance‐phase spinal hub increase as a robust network phenomenon while refraining from strong causal claims about its origin. Future experiments combining spinal cord transection, optogenetic silencing of CPG circuits, or dorsal rhizotomy with simultaneous cortical and spinal recording would be required to dissociate these contributions.

Anatomical evidence from corticospinal tract (CST) studies provides a structural framework for interpreting our functional findings. CST projections from the hindlimb M1 region terminate in the lumbar spinal cord and are essential for coordinated locomotion [48]; approximately 90% of CST fibers project contralaterally, with the remaining 10% projecting ipsilaterally to mediate bilateral limb coordination [49]. However, it is important to emphasize that anatomical connectivity is not equivalent to functional connectivity: The existence of a synaptic pathway does not directly predict the strength, timing, or state‐dependence of the functional coupling observed in any given behavioral context. The GC‐ and TSPE‐derived connectivity metrics reported here reflect the statistical dynamics of neural co‐activity during locomotion and are modulated by factors including synaptic efficacy, neuromodulatory tone, and behavioral phase—none of which are captured by anatomical tract‐tracing data alone.

In the present study, recording electrodes were implanted in the contralateral M1 and the ipsilateral lumbar spinal cord (relative to the dominant hindlimb), a configuration that preferentially captures corticospinal communication through both the contralateral CST projection (via decussation) and potential ipsilateral pathways. Our experimental design did not include simultaneous bilateral recordings, and therefore we cannot directly test whether contralateral CST‐mediated corticospinal coupling dominates over ipsilateral projections in the functional connectivity metrics reported. This represents a limitation that future studies employing bilateral cortical and spinal recording arrays should address.

### 4.3 Implications for Spinal Cord Injury and Rehabilitation

The development of chronic multichannel recording systems in awake, freely moving animals has enabled detailed exploration of neural dynamics in health and disease. The observation that spinal hub neurons exhibit shorter transmission latency and stronger beta coupling during stance suggests that these neurons may represent functionally privileged targets for neuromodulation intervention. Spinal cord stimulation (SCS) applied to hub‐enriched lumbar regions has shown efficacy in restoring locomotion in rodent SCI models [50] and in human patients [51, 52], lending plausibility to the hypothesis that hub‐targeted stimulation could enhance rehabilitative outcomes. However, this remains an inference: The present study did not test stimulation effects, and the causal relationship between hub neuron activity and locomotor recovery has not been established.

In SCI models, cortical motor maps undergo plastic changes, often mediated by reticulospinal circuits [53, 54]. Compared to rodents, damage to the corticospinal system is more detrimental in humans due to our more specialized cortico‐motoneuronal pathways [43, 55]. Recent studies have shown that electrochemical stimulation of lumbar circuits can activate glutamatergic reticulospinal neurons and reconfigure cortical connectivity in injured systems. Simultaneous recording at both cortical and spinal levels, as used in this study, will be essential for characterizing these adaptive changes.

### 4.4 Potential of Network Analysis in Neurorehabilitation

Our findings offer valuable insights into the use of network analysis for designing targeted neuromodulation strategies. The normative corticospinal network topology characterized in this study—including phase‐specific global efficiency, hub neuron distribution, and beta‐band coupling patterns—provides a quantitative reference framework against which pathological disruptions can be compared. Network analysis approaches such as TSPE‐based hub identification and GC‐derived connectivity matrices are directly applicable to recordings obtained from injured or disease‐model animals, enabling systematic comparison of pre‐ and post‐injury network reorganization. This analytical framework is a direct and immediate contribution of the present work. Future research should explore how these networks evolve over time post‐injury and assess the therapeutic potential of spinal cord stimulation (SCS) [56, 57] and other neuromodulation approaches.

## 5 Limitations

Several limitations of the present study should be acknowledged to appropriately contextualize the findings.

### 5.1 Small Sample Size

Only four animals were included in this study. While the within‐subject repeated‐measures design and the large number of simultaneously recorded single units (total: 33 cortical and 27 spinal neurons) provide reasonable statistical power for unit‐level analyses, the animal‐level sample size of *n* = 4 limits the generalizability of findings and reduces the sensitivity of between‐animal variability estimates. Replication in larger cohorts is needed to confirm the robustness of the reported network metrics, particularly the rich‐club coefficients whose variance was substantial across phases.

### 5.2 Non‐Natural Locomotor Behavior

Bipedal walking in rats is not a spontaneous behavior; it was acquired through treadmill training under body weight support via a restraint jacket. This paradigm introduces biomechanical and postural demands—particularly active trunk stabilization—that are absent in natural quadrupedal locomotion and differ from human bipedal gait in important respects. The corticospinal network dynamics reported here may therefore reflect a mixture of hindlimb locomotor control and postural stabilization circuitry, and may not fully generalize to unrestrained quadrupedal locomotion or to the neural control of human walking.

#### 5.2.1 Electrode‐Induced Tissue Effects

Implantation of rigid microwire electrodes into the spinal cord inevitably causes localized tissue displacement, glial scarring, and potential disruption of intra‐spinal microcircuits in the vicinity of the electrode. These effects, which evolve over the weeks following implantation, may alter the functional connectivity of recorded neurons relative to the intact state. Although recordings began at least one week post‐surgery to allow initial recovery, chronic electrode‐tissue interactions cannot be fully excluded as a confound.

#### 5.2.2 Spike Waveform Stability Verification

It should be noted that spike waveform stability across sessions was not directly verified using template cross‐correlation analysis in the present study. The > 20% impedance change exclusion criterion served as an indirect proxy for electrode stability. Future studies should incorporate direct waveform shape verification (e.g., Pearson cross‐correlation between pre‐ and post‐recording spike templates, with *r* < 0.90 as an exclusion threshold) to more rigorously confirm single‐unit identity across recording phases.

### 5.3 Statistical Versus Synaptic Connectivity

Both TSPE‐based functional coupling and Granger causality are statistical measures derived from the temporal co‐variation of neural signals. They do not directly measure synaptic connectivity, neurotransmitter identity, or the physical presence of axonal projections. A statistically significant TSPE edge between a cortical and a spinal neuron indicates temporally correlated spiking consistent with a functional relationship, but does not confirm monosynaptic connectivity, rule out common‐input confounds, or establish the sign of the synaptic interaction with certainty. Interpretations of TSPE and GC results as reflections of “effective” or “functional” connectivity should be understood within these methodological constraints.

### 5.4 Absence of Causal Intervention

This study is observational in design: Corticospinal network properties were characterized during naturally occurring locomotor states, but no experimental manipulation (e.g., pharmacological silencing, optogenetic inhibition, spinal cord injury, or neuromodulatory stimulation) was performed to test causal relationships. Consequently, while our data are consistent with a model in which spinal hub neurons actively coordinate locomotion, they cannot exclude the possibility that the observed hub structure is an epiphenomenon of sensory input or CPG activity rather than a causal driver of motor output. Future studies incorporating targeted circuit perturbations are essential to establish mechanistic causality.

### 5.5 Electrode Coverage and Regional Representativeness

Spinal recordings were confined to the L1–L2 segment, which primarily represents hip muscle motor pools. Neurons controlling the knee, ankle, and foot—located at L3–S1—were not directly sampled. The characterization of “spinal corticospinal network hubs” is therefore specific to the lumbar enlargement hip circuitry and may not represent the full spatial extent of locomotor spinal network organization.

## 6 Conclusion

To our knowledge, this is the first study to simultaneously record single‐unit spiking activity from both the hindlimb primary motor cortex (M1) and the lumbar spinal cord in rats performing bipedal locomotion, and to characterize the resulting corticospinal functional networks using TSPE‐based spike‐train connectivity analysis in conjunction with graph‐theoretical metrics. Three quantitatively supported findings emerge from this work. First, corticospinal network global efficiency increased significantly from rest (0.33) to stance (0.53) and swing (0.51), indicating that active locomotion drives a more integrated inter‐regional communication architecture. Second, the proportion of spinal rich‐club hub neurons—defined at the level of individual high‐degree neurons rather than gross anatomical regions—rose from 55.48% at rest to 81.13% during stance (*p* = 0.028), demonstrating a phase‐dependent shift in network centrality toward spinal circuits during weight‐bearing that partially reversed during swing (60.48% spinal). Third, mean corticospinal signal transmission latency decreased significantly from 29.60 ± 20.66 ms at rest to 9.67 ± 1.65 ms during stance (*p* = 0.001), reflecting accelerated inter‐regional signaling during the weight‐bearing phase. These findings are reported as observations at the functional network level. The causal mechanisms underlying the stance‐phase hub shift—whether primarily driven by descending motor commands, peripheral sensory feedback, or intrinsic spinal CPG dynamics—cannot be resolved from the present correlational data and require future intervention‐based investigation.

The corticospinal network reorganization documented here extends prior neuroimaging‐based descriptions of static spinal cord architecture by capturing millisecond‐scale, single‐neuron dynamics during active locomotion. The preferential enhancement of beta‐band (13–35 Hz) corticospinal effective connectivity during stance, combined with high‐gamma (50–150 Hz) concentration of local neuronal energy during movement, suggests a functional dissociation between inter‐regional command transmission and local motor execution—a distinction that cannot be resolved by neuroimaging approaches. The normative TSPE‐derived connectivity atlas established here provides a quantitative baseline against which pathological disruptions of corticospinal networks—as occur in spinal cord injury, Parkinson's disease, and stroke—can be systematically characterized in future studies using the same recording and analytical framework.

The translational implications of the present findings are appropriately constrained by the observational, healthy‐animal design. The identified phase‐specific beta‐band coupling patterns and spinal hub neuron dynamics represent candidate network‐level biomarkers whose utility for guiding closed‐loop neuromodulation in injured or disease‐affected systems remains to be tested experimentally. The normative framework established in this study provides the necessary reference for such future investigations and ultimately defines the corticospinal network architecture that rehabilitation interventions aim to restore.

## Author Contributions

Author contributions: Data curation, Pengcheng Xi and Yiran Lang; Formal analysis, Pengcheng Xi and Xiaodan Lyu; Funding acquisition, Jiping He; Methodology, Pengcheng Xi and Xiaodan Lyu; Project administration and supervision, Yiran Lang; Writing – original draft, Pengcheng Xi; Writing – review and editing, Rongyu Tang and Yiran Lang. All authors have read and agreed to the published version of the manuscript.

## Funding

This work was supported by the National Natural Science Foundation of China (82371394) and the National Natural Science Foundation of China (U2541230).

## Ethics Statement

All experimental procedures were approved by the Ethics Committee of the university (Approval No. BIT‐EC‐SCXK‐2019‐0010‐R‐2020037) and conducted in accordance with institutional and national guidelines. Reporting adhered to the ARRIVE (Animal Research: Reporting of In Vivo Experiments) guidelines.

## Conflicts of Interest

The authors declare no conflicts of interest.

## Supporting information


**Figure S1:** Average degree for Rat 1.
**Figure S2:** Global efficiency for Rat 1.
**Figure S3:** Clustering coefficient for Rat 1.
**Figure S4:** Average degree for Rat 2.
**Figure S5:** Global efficiency for Rat 2.
**Figure S6:** Clustering coefficient for Rat 2.
**Figure S7:** Average degree for Rat 3.
**Figure S8:** Global efficiency for Rat 3.
**Figure S9:** Clustering coefficient for Rat 3.
**Figure S10:** Rich‐club node proportion across rest, stance, and swing phases.
**Figure S11:** Connection proportion across rest, stance, and swing phases.

## Data Availability

The data that support the findings of this study are available from the corresponding author upon reasonable request.
