# Supplementary material for: Neural Network Connection Characteristics Between Motor Cortex and Spinal Cord During Rat Bipedal Walking
Source: CNS Neurosci Ther. 2026 Sep 18;32(9):e71160. doi: 10.1002/cns.71160 (PMC13588150; doi:10.1002/cns.71160)
Supplement: Supplementary file 1 [file CNS-32-e71160-s001.docx]

**Supplementary Materials**

*Neural Network Connection Characteristics between Motor Cortex and Spinal Cord During Rat Bipedal Walking*

**Supplementary Figures**

The following supplementary figures provide animal-level validation for the additional three animals. Figures S1-S9 show average degree, global efficiency, and clustering coefficient. Figures S10-S11 show rich-club node proportion and connection proportion results.

Supplementary Figure S1. Average degree for Rat 1

Supplementary Figure S2. Global efficiency for Rat 1

Supplementary Figure S3. Clustering coefficient for Rat 1

Supplementary Figure S4. Average degree for Rat 2

Supplementary Figure S5. Global efficiency for Rat 2

Supplementary Figure S6. Clustering coefficient for Rat 2

Supplementary Figure S7. Average degree for Rat 3

Supplementary Figure S8. Global efficiency for Rat 3

Supplementary Figure S9. Clustering coefficient for Rat 3

Supplementary Figure S10. Rich-club node proportion across rest, stance, and swing phases.

Supplementary Figure S11. Connection proportion across rest, stance, and swing phases.
